# New Insights on the Mechanism of Quinoline-based DNA Methyltransferase Inhibitors*

**DOI:** 10.1074/jbc.M114.594671

**Published:** 2014-12-18

**Authors:** Christina Gros, Laurence Fleury, Virginie Nahoum, Céline Faux, Sergio Valente, Donatella Labella, Frédéric Cantagrel, Elodie Rilova, Mohamed Amine Bouhlel, Marie-Hélène David-Cordonnier, Isabelle Dufau, Frédéric Ausseil, Antonello Mai, Lionel Mourey, Laurent Lacroix, Paola B. Arimondo

**Affiliations:** From the ‡ Unité de Service et de Recherche CNRS-Pierre Fabre 3388, ETaC, CRDPF, 31100 Toulouse, France,; § Institut de Pharmacologie et de Biologie Structurale (IPBS) CNRS, Toulouse, 31077, France,; ¶ Université de Toulouse, UPS, IPBS, Toulouse, 31077, France,; ‖ Sapienza University of Rome, Department of Chemistry and Technology of Drug, Sapienza University of Rome, I-00185 Roma, Italy,; ** INSERM UMR837-JPARC (Jean-Pierre Aubert Research Center), Team 4, IRCL, 59045 Lille, France,; ‡‡ Pasteur Institute-Cenci Bolognetti Foundation, Sapienza University of Rome, I-00185 Roma, Italy, and; §§ CNRS UMR 5099, LBME, 31062 Toulouse, France

**Keywords:** DNA, DNA Methyltransferase, DNA-Protein Interaction, Enzyme Inhibitor, Gene Regulation, SGI-1027, Competition, Inhibition Mechanism

## Abstract

Among the epigenetic marks, DNA methylation is one of the most studied. It is highly deregulated in numerous diseases, including cancer. Indeed, it has been shown that hypermethylation of tumor suppressor genes promoters is a common feature of cancer cells. Because DNA methylation is reversible, the DNA methyltransferases (DNMTs), responsible for this epigenetic mark, are considered promising therapeutic targets. Several molecules have been identified as DNMT inhibitors and, among the non-nucleoside inhibitors, 4-aminoquinoline-based inhibitors, such as SGI-1027 and its analogs, showed potent inhibitory activity. Here we characterized the *in vitro* mechanism of action of SGI-1027 and two analogs. Enzymatic competition studies with the DNA substrate and the methyl donor cofactor, *S*-adenosyl-l-methionine (AdoMet), displayed AdoMet non-competitive and DNA competitive behavior. In addition, deviations from the Michaelis-Menten model in DNA competition experiments suggested an interaction with DNA. Thus their ability to interact with DNA was established; although SGI-1027 was a weak DNA ligand, analog 5, the most potent inhibitor, strongly interacted with DNA. Finally, as 5 interacted with DNMT only when the DNA duplex was present, we hypothesize that this class of chemical compounds inhibit DNMTs by interacting with the DNA substrate.

## Teaser

**Background:** 4-Aminoquinoline SGI-1027 and analogs inhibit DNA methylation, which is deregulated in cancers.

**Results:** These compounds induce deviations from Michaelis-Menten equations in DNA competition experiments and interact with DNA.

**Conclusion:** They are competitive inhibitors for the DNA substrate of the DNA methyltransferase and non-competitive for the methyl group donor, *S*-adenosyl-l-methionine.

**Significance:** These findings suggest a mechanism of inhibition for these 4-aminoquinoline-based DNMT inhibitors.

## Introduction

Methylation of cytosine residues in DNA, occurring in humans at CpG sites, is an important epigenetic mark highly deregulated in cancer (1). Indeed, hypermethylation of tumor suppressor genes promoters is a common feature of cancer cells and leads to abnormal silencing of the targeted genes. Because it is a reversible phenomenon, DNA methyltransferases (DNMTs),^3^ which catalyze the transfer of a methyl group from *S*-adenosyl-l-methionine (AdoMet) to the C5-position of cytosine, are promising therapeutic targets (2).

DNMTs are divided into two categories: DNMT1, which is mainly involved in the maintenance of the methylation pattern and is active on hemimethylated DNA, and DNMT3s, which are active both on unmethylated and hemimethylated substrates, are responsible of *de novo* DNA methylation, and include DNMT3A, DNMT3B, and the catalytically inactive DNMT3L (3).

Several inhibitors of these enzymes have been described and reviewed in numerous publications (4–6). The nucleoside analogs azacitidine (Vidaza) and decitabine (Dacogen) have been approved by the Food and Drug Administration in 2004 and 2006, respectively, for hematological malignancies, whereas some other nucleoside-like analogs are currently in clinical trials in hematological diseases and solid tumors (5, 7, 8). However, their poor bioavailability, their chemical instability in physiological media, and their lack of selectivity reveal an urgent need for novel, more selective and non-nucleoside inhibitors. Among these, various inhibitors have been characterized, but most of them are nonspecific and/or do not induce DNA demethylation in cells (5, 6), except for **SGI-1027**, a quinoline derivative that was described by Datta *et al.* in 2009 (9) for its enzymatic and cellular DNMT inhibition.

Initially synthesized as part of a minor-groove binders family of quinolinium bisquaternary salts, **SGI-1027** inhibits bacterial DNA methyltransferase *M.SssI*, human DNMT1, mouse Dnmt3A, and mouse Dnmt3B (9). It is currently the reference compound in several DNMT inhibition assays (10, 11) and structure-activity relationship studies (12). Therefore, there is an actual interest in elucidating its molecular mechanism of action.

**FIGURE 1.**
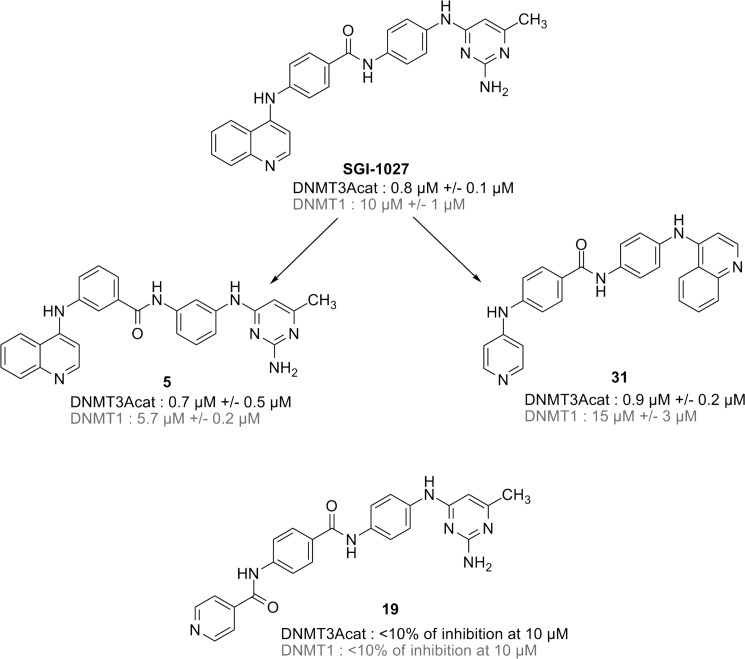
**Chemical structures and enzymatic activities of SGI-1027 and its analogs.** The IC_50_ against DNMT3Acat and DNMT1 are reported. For **19**, the percentages of inhibition of DNMT3Acat or DNMT1 are displayed. The means of two experiments with the corresponding S.E. are shown. The compounds were named accordingly to the nomenclature of the respective articles.

Two groups performed competition studies on DNMT1 (9, 10) and concluded that **SGI-1027** was a AdoMet-competitive and DNA non-competitive inhibitor of DNMT1. Here, we studied the mechanism of inhibition of full-length human DNMT1 by **SGI-1027** and two analogs that we recently synthesized (compounds **5** and **31** in Fig. 1, described by Valente *et al.* (13) and Rilova *et al.* (14), respectively). In contrast to previously reported data (9, 10), our findings clearly support a behavior as DNA competitive and AdoMet non-competitive inhibitors. The ability of the compounds to interact with DNA and DNMT1 was investigated to further characterize the mechanism of action using compound **19** (Fig. 1) as a negative control as it did not succeed to inhibit either DNMT1 or human catalytic DNMT3A (DNMT3Acat) (14). Several hypotheses are described, and the differences with the literature are discussed.

## EXPERIMENTAL PROCEDURES

### 

#### 

##### General

All commercially available reagents and solvents were purchased from Sigma, and radioactive [methyl-^3^H]AdoMet was from PerkinElmer Life Sciences. **SGI-1027**, compounds **19** and **31**, and compound **5** were synthesized as described in Refs. 9, 14, and 13, respectively. 10 mm stock solutions were prepared in DMSO and aliquoted. The compounds were named according to the nomenclature of the respective articles.

##### Enzyme Production

Full-length histidine-tagged human DNMT1 (182 kDa) was produced and purified according to Lee *et al.* (15). Catalytic human DNMT3Acat (DNMT3Acat: residues 623–908 amino acids) was produced and purified according to Gros *et al.* (16).

##### DNMT Inhibition Assays

DNMT1 inhibition assay was developed and described in Gros *et al.* (16). DNMT3Acat inhibition was described in Rilova *et al.* (14).

##### DNMT1 Competition Assays

Competition assays on full-length DNMT1 were realized according to Gros *et al.* (16). Briefly, the tested compound, biotinylated duplex, [*methyl*-^3^H]AdoMet and DNMT1 were incubated for 2 h in 10 μl at 37 °C. An aliquot of 8 μl was then transferred in a Flashplate^TM^ well containing 190 μl of 20 μm SAH solution in Tris-HCl. The Flashplate^TM^ was agitated at room temperature for 1 h, washed 3 times with 200 μl of 0.05% Tween® 20 in Tris-HCl, and read in 200 μl of Tris-HCl on TopCount NXT.

In AdoMet competition assays, [methyl-^3^H]AdoMet was varied between 0.5 and 15 μm at a fixed DNA duplex concentration of 0.6 μm. For each AdoMet concentration the tested compound concentration was adjusted between its IC_10_ and IC_80_. For each compound concentration, the Michaelis-Menten model was fitted by non-linear regression to the data, and *K*_*m*_^app^ and V_m_^app^ were calculated according to this model. For Lineweaver-Burk plots, a linear model was fitted by linear regression to the transformed data. Lineweaver-Burk or double-reciprocal plots were only used as a graphic representation to distinguish competitive, non-competitive, and uncompetitive inhibitor. Noteworthy, this graphic representation is known to distort and magnify the errors of the data.

In DNA-competition assays, the DNA duplex concentration was varied between 0.05 and 0.6 μm, whereas [*methyl*-^3^H]AdoMet concentration was held at 15 μm. For each DNA duplex concentration, the tested compound concentration was adjusted between its IC_10_ and IC_80_ values. For each compound concentration, the Copeland and Horiuchi (17) non-competitive, uncompetitive, and competitive models were fitted by non-linear regression to the data. For each molecule, the only convergent model at each concentration was the Copeland and Horiuchi (17) competitive model. In the Lineweaver-Burk plots, the double reciprocal of the Copeland and Horiuchi competitive model (17) was fitted by non-linear regression to the transformed data.

In both AdoMet and DNA competition assays, for each substrate concentration, the IC_50_ of the tested compound was calculated by non-linear regression fitting with sigmoidal dose-response (variable slope) with constrained top and bottom at 100 and 0% of inhibition, respectively. All the non-linear and linear regressions were performed on GraphPad Prism 4.03 (GraphPad Software).

##### T_m_ Assay

DNA thermal denaturation experiments were conducted as described in Mergny and Lacroix (18). Hairpin DNA duplexes hp_2_CG (5′-TATATACGTACGGTGTTTTCACCGTACGTATATA-3′) containing 2 CpG sites, hp_1_CG (5′-TATATACGTACTGTGTTTTCACAGTACGTATATA-3′) containing 1 CpG site, and hp_0_CG (5′-TATATATGTACTGTGTTTTCACAGTACATATATA-3′) containing no CpG site were used at 2 μm in the absence or presence of the inhibitor in the *T_m_* assay buffer (100 mm NaCl, lithium cacodylate 20 mm, pH 7.2). The temperature at which 50% of the duplex is denatured, *T_m_*, was calculated as previously described (18). Means of at least two experiments with the corresponding S.E. are reported.

##### DNase I Footprinting

DNase I footprinting experiments were performed essentially as described in Lemster *et al.* (19) and Racané *et al.* (20). Briefly, the 117- and 265-bp DNA fragments were obtained from EcoRI and PvuII double digestion of the pBS plasmid (Stratagene, La Jolla, CA). The generated DNA fragments was 3′-end-labeled for 30 min at 37 °C using 10 units of Klenow enzyme (New England BioLabs) and [α-^32^P]dATP (3000Ci/mmol, PerkinElmer Life Sciences) before isolation on a 6% polyacrylamide gel under native conditions. The radiolabeled 117- and 265-bp DNA fragments were cut off from the gel, crushed, dialyzed overnight against 400 μl of elution buffer (10 mm Tris-HCl, pH 8.0, 1 mm EDTA, 100 mm NaCl), and then separated from polyacrylamide gel by filtration through a Millipore 0.22-μm membrane followed by ethanol precipitation. Appropriate concentrations of the various tested compounds were incubated with the 117- or 265-bp radiolabeled DNA fragments for 15 min at 37 °C to ensure equilibrium before the addition of 1 unit/μl of DNase I in appropriate buffer for 3 min of digestion. The reaction was stopped by ethanol precipitation. The digested DNAs were subsequently dissolved in 4 μl of denaturing loading buffer (80% formamide solution containing tracking dyes), heated for 4 min at 90 °C, and chilled 4 min on ice before electrophoresis for 90 min at 65 watts on a 8% denaturing polyacrylamide gel in Tris/borate/EDTA buffer. Finally, gels were soaked in 10% acetic acid, transferred to Whatman No. 3MM paper to be dried under vacuum at 80 °C, and exposed overnight at room temperature on phosphor-imaging storage screens. The identity of the bases from each DNA fragment was established from comparison of the relative position of the bands to the guanine sequencing standard (G-track) classically obtained using dimethyl sulfate and piperidine treatment of the same DNA fragment.

##### Differential Scanning Fluorimetry Assay

Experiments were conducted using a CFX384^TM^ Real-Time System (C1000 Thermal cycler, Bio-Rad CFX Manager 2.0 Software, Bio-Rad). The samples were heated at 0.1 °C/s, from 10 to 80 °C. The fluorescence intensity was plotted as a function of the temperature. *T*_½_ was given by the inflection point of the fluorescence curve. Δ*T*_½_ was calculated by subtracting the *t*_½_ in the absence of the compound to the *T*_½_ in the presence of the compound in the same condition (*i.e.* in the presence of other partners DNA and/or AdoMet).

The protein was scanned to assess suitability of the method, and the lowest concentration of DNMT1 protein needed to generate a strong signal was determined to be 2.5 μm. Compound concentrations varied between 5 and 200 μm. The DNA duplex used in the enzymatic assays was chosen and added at 5 μm. The AdoMet cofactor was added at a final concentration of 5 μm or not. The SYPRO orange dye (Invitrogen) was diluted to 1/400th in each sample. Each experiment was repeated for at least two times in duplicate. Means of at least two experiments are displayed with the corresponding S.E.

##### DNA Duplex and DNMT1 Gel Shift Assays

4 μl of compounds or 1% of DMSO, 0.5 μm 6-carboxyfluorescein 5′-labeled DNA duplex used in the DNMT1 enzymatic assay, and 5 μm AdoMet were added in each well and incubated for 30 min at 37 °C with or without 2.5 μm DNMT1. Samples were loaded on a 0.7% agarose gel and migrated for 50 min at 135 V in 1× Tris/borate/EDTA electrophoresis buffer. Gels were analyzed with the Typhoon trio® of GE Healthcare with appropriated filters.

## RESULTS

### 

#### 

##### SGI-1027 and Its Analogs Share DNA-competitive and AdoMet Non-competitive Behavior on DNMT1

We recently developed a new DNMT1 inhibition assay in homogenous phase that allows to study the mechanism of action of potent inhibitors of DNMT1 by carrying out enzymatic studies (16). We decided here to apply it to the study of 4-aminoquinoline **SGI-1027**, a well described inhibitor of DNMTs, and two analogs that we have recently synthesized, **31** and **5** (Fig. 1) (13, 14). Interestingly, the latter is a position isomer of **SGI-1027**, in which the aminopyrimidine and the phenyl group are attached in *meta* of the amide function and not in *para*, as in **SGI-1027** (Fig. 1). Derivative **31** contains a pyridine instead of the pyrimidine cycle and an inverted amide at the center. The three compounds are potent inhibitors of DNMT1 (IC_50_ of 10 μm for **SGI-1027**, 15 μm for **31**, and 5.7 μm for **5**, Fig. 1 and Table 1) and of DNMT3Acat (IC_50_ of 0.8, 0.9, and 0.7 μm, respectively; Fig. 1 and Table 1) (14). The assay that we developed was particularly suitable to carry out competition experiments on DNMT1, which methylates DNA with higher yields than DNMT3Acat. In addition, we dispose of the full-length human DNMT1, containing all the important domains for the interaction of the enzyme with DNA and with the inhibitors (21), whereas we only disposed of the C-terminal domain of DNMT3A (DNMT3Acat). Therefore, full-length DNMT1 was chosen to perform all further competition experiments.

**TABLE 1:** **DNMT1 and DNMT3Acat inhibition activity of SGI-1027 and its analogs and differences in the *T_m_* values of the duplexes in the presence and in the absence of the compounds (Δ*T_m_*)** The concentrations (μm) at which 50% of the methylation activity is inhibited (IC_50_) are reported for DNMT3Acat and DNMT1. For **19**, the percentages of inhibition are displayed. The difference in *T_m_* (Δ*T_m_* in °C) in the presence of 10 μm of inhibitors and in their absence is reported for each duplex. Means of at least two experiments with the corresponding standard errors are reported. The means of measured *T_m_* are displayed in parentheses (°C).

Compounds	IC_50_		Δ*T_m_* (measured *T_m_* in °C) at 10 μm		
	*DNMT3Acat*	*DNMT1*	*hp_2_CG*	*hp_1_CG*	*hp_0_CG*
	μ*m*		°C		
**SGI-1027**	0.8 μm ± 0.1 μm	10 μm ± 1 μm	−0.5 ± 0.0 (74.0)	1.0 ± 0.0 (72.0)	0.5 ± 0.0 (68.5)
**5**	0.7 μm ± 0.5 μm	5.7 μm ± 0.2 μm	1.5 ± 0.0 (76.0)	3.0 ± 0.0 (74.0)	4.0 ± 0.0 (72.0)
**31**	0.9 μm ± 0.2 μm	15 μm ± 3 μm	0.3 ± 0.3 (74.8)	1.1 ± 0.2 (72.1)	1.6 ± 0.4 (69.6)
**19**	<10% of inhibition at 10 μm	<10% of inhibition at 10 μm	0.5 ± 0.0 (75.0)	0.5 ± 0.0 (71.5)	0.3 ± 0.3 (68.3)

**FIGURE 2.**
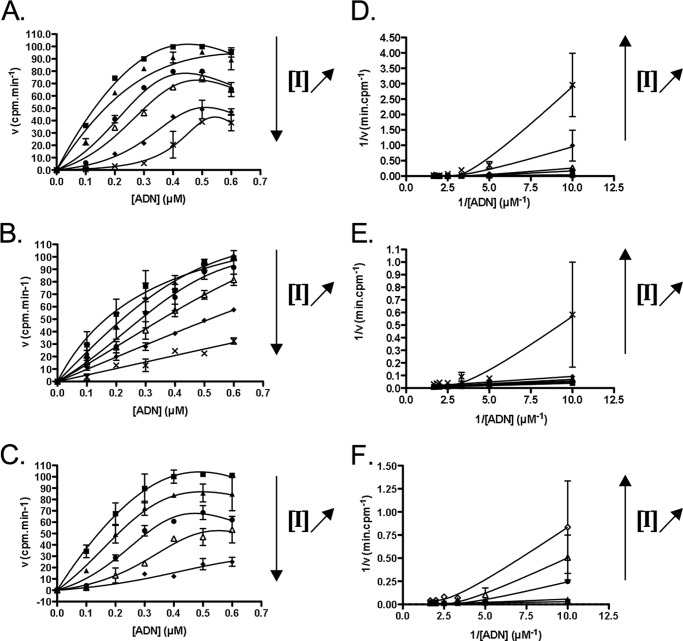
**DNA-competition studies with SGI-1027 and its analogs in a DNMT1 enzymatic assay.** *A–C*, initial velocity plots against DNA concentrations for different concentrations of **SGI-1027** (*A*), **5** (*B*), and **31** (*C*). *Squares* represent points in the absence of the inhibitor. For **SGI-1027** (*A*): *triangles*, *circles*, *empty triangles*, *diamonds*, and *crosses* represent 5.6, 10, 14.7, 21.5, and 32 μm, respectively. For **5** (*B*): *triangles*, *circles*, *empty triangles*, *diamonds*, and *crosses* represent 3.2, 4.2, 5.6, 7.5, and 10 μm, respectively. For **31** (*C*): *triangles*, *circles*, *empty triangles*, and *diamonds* represent 10, 25, 40, and 55 μm, respectively. *Plain lines* represent fittings with the Copeland and Horiuchi (17) competitive model. *D–F*, Lineweaver-Burk (or double reciprocal) plots for **SGI-1027** (*D*), **5** (*E*), and **31** (*F*). For the corresponding concentrations, the same legend is applied as for *A–C. Plain lines* represent fittings with the double-reciprocal of Copeland and Horiuchi (17) competitive model. The means of two experiments are displayed with the corresponding S.E.

First, we studied the behavior of the inhibitors to compete with the DNA substrate of the enzyme in the DNMT1 enzymatic test. To analyze whether the compounds were DNA competitors or not, we performed non-linear regression with the three models (competitive, non-competitive, and uncompetitive inhibition) described by Copeland and Horiuchi (17). Interestingly, DNA competition studies displayed unexpected results (Fig. 2). Velocity plots against substrate concentration did not follow the Michaelis-Menten behavior and presented a sigmoidal character that was particularly significant at high inhibitor concentrations (Fig. 2, *A–C*). This phenomenon resulted in a deformation of the Lineweaver-Burk plots (Fig. 2, *D–F*). Indeed, at high inhibitor concentrations, points no longer displayed a linear behavior but rather an up-turning parabolic character that could be approximated with a quadratic function. Copeland and Horiuchi (17) explained these features as being characteristic of competitive inhibitors that might interact with DNA.

In agreement with these competitive behaviors (22), the measured IC_50_ of the compounds increased as the DNA concentration increased (data not shown). However, in the case of substrate-inhibitor interactions, the IC_50_ analysis is not sufficient to conclude whether **SGI-1027** and its analogs are DNA competitors or not (17). Thus, velocity plots, double-reciprocal plots, and IC_50_ against [DNA]/*K*_*m*_^DNA^ plots were analyzed; all suggested a DNA competitive inhibition of DNMT1 (Fig. 2 and data not shown). Moreover, concerning compound **5**, the sigmoid is so stretched to appear as aligned points (Fig. 2*B*). This is a mathematical limit of the Copeland and Horiuchi (17) model that occurs when nearly all the inhibitor in solution is complexed to the substrate, in our case the DNA, leading to an apparent straight line. This suggested that compound **5** is a stronger DNA binder than **31** and **SGI-1027**.

**FIGURE 3.**
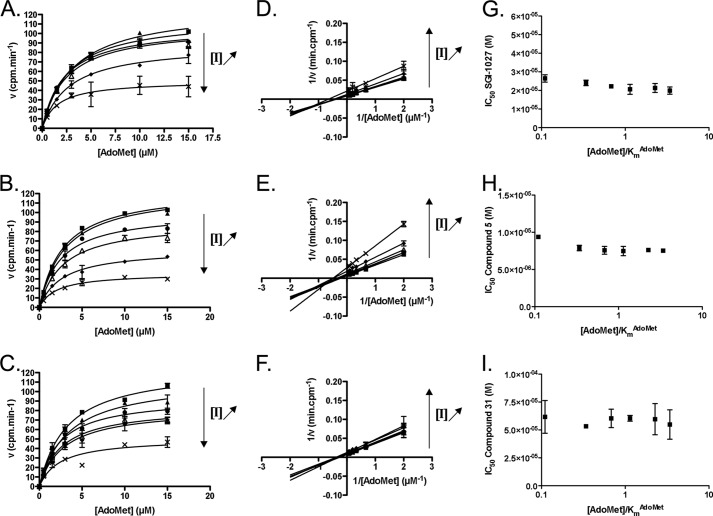
**AdoMet competition studies of SGI-1027 and its analogs in a DNMT1 enzymatic assay.** *A–C*, initial velocity plots against AdoMet concentrations for different concentrations of **SGI-1027** (*A*), **5** (*B*), and **31** (*C*). *Squares* represent points without inhibitor. For **SGI-1027** (*A*): *triangles*, *circles*, *empty triangles*, *diamonds*, and *crosses* represent 3.2, 5.6, 10, 14.7, and 21.5 μm, respectively. For **5** (*B*): *triangles*, *circles*, *empty triangles*, *diamonds*, and *crosses* represent 3.2, 4.2, 5.6, 7.5, and 10 μm, respectively. For **31** (*C*): *triangles*, *circles*, *empty triangles*, *diamonds*, and *crosses* represent 3.2, 5.6, 10, 20, and 32 μm, respectively. *Plain lines* represent fittings with the Michaelis-Menten model. *D–F*, Lineweaver-Burk (double reciprocal) plots for **SGI-1027** (*D*), **5** (*E*), and **31** (*F*). For the corresponding concentrations, the same legend is applied as for *A–C. Plain lines* represent fittings with the linear model to the data. *G–I*, IC_50_ of **SGI-1027** (*G*), **5** (*H*), and **31** (*I*), respectively, as a function of [AdoMet]/*K*_*m*_^AdoMet^. As the fittings according to a linear model were not significantly different from zero (*p* < 0.05), they were not displayed. The means of two experiments are displayed with the corresponding S.E.

In contrast, in AdoMet competition studies, each compound presented a Michaelis-Menten behavior with hyperbolic velocity plots against the substrate concentration (Fig. 3, *A–C*). Moreover, this was confirmed by the double-reciprocal plots (Fig. 3*D*-F). As the *K*_*m*_^app^ remained constant and the 1/V_m_^app^ increased with increasing inhibitor concentrations (data not shown), **SGI-1027** and its analogs resulted in AdoMet non-competitive inhibitors of DNMT1. In addition, the overall unchanged IC_50_ regardless of the AdoMet concentration confirmed this hypothesis (Fig. 3, *G–I*) (22).

Thus, our results showed that **SGI-1027** and its analogs display AdoMet non-competitive behaviors in agreement with the Michaelis-Menten model in experimental conditions in which the DNA concentration is constant. On the other hand, the three compounds seemed to be DNA competitors with deviations from the Michaelis-Menten equation at high inhibitor concentrations. As this phenomenon might be characteristic of the inhibitor interacting with the DNA, we next evaluated this feature by using parent compound **19** as a negative control as it inhibits neither DNMT1 nor DNMT3Acat.

##### **SGI-1027** Interacts Weakly with DNA, and Compound **5** Binds Strongly to DNA

**FIGURE 4.**
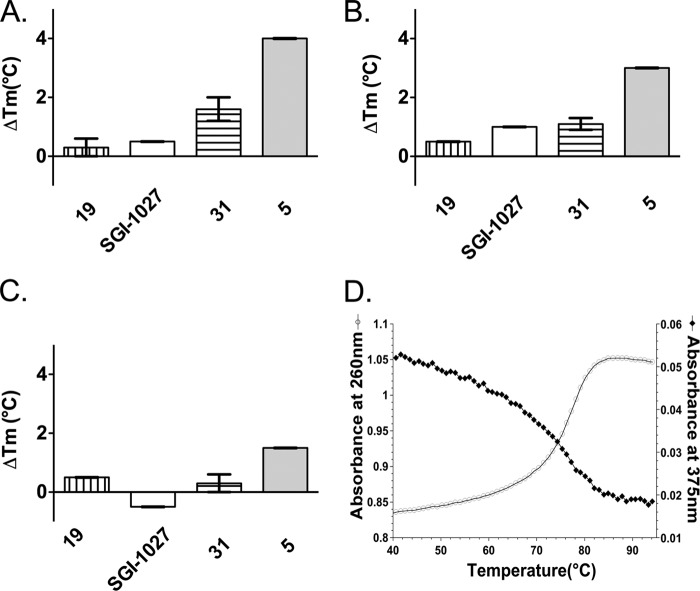
**Δ*T_m_* value for the hairpin duplexes in the presence of SGI-1027, compound 19, 31 and 5.** *A–C*, each duplex (hp_0_CG, *panel A*; hp_1_CG, *panel B*; hp_2_CG, *panel C*) was incubated with 10 μm **19** (*vertically stripped bars*), **SGI-1027** (*white bars*), **31** (*horizontally stripped* bars), and **5** (*gray bars*). The mean of at least two experiments is reported with the corresponding S.E. *D*, the absorbance of the bases at 260 nm (*white circles*) and of compound **5** at 375 nm (*black diamonds*) is recorded for the duplex in the presence of compound **5** as a function of the temperature (°C).

To study the interaction between the compounds and DNA, we followed thermal denaturation of short DNA duplexes by UV absorbance in the absence and in the presence of the compounds. Three hairpin duplexes were chosen containing no CpG site (hp_0_CG, *T_m_* = 68.0 °C), 1 CpG site (hp_1_CG, *T_m_* = 71.0 °C), and 2 CpG sites (hp_2_CG, *T_m_* = 74.5 °C). Fig. 4, *A–C*, and Table 1 report the differences observed in *T_m_* (Δ*T_m_*) for the DNA duplexes when in the presence of **19**, **SGI-1027**, **31**, and **5**. No significant increase in the *T_m_* value was observed when the negative control **19** was incubated with any of the DNA duplexes, whereas **SGI-1027** showed a slight increase of the *T_m_* value when incubated with hp_1_CG (Δ*T_m_* = 1.0 °C). The data with compounds **31** showed a higher S.E. because the molecule was less soluble in DMSO. It showed a concentration effect on the *T_m_* of the duplexes (data not shown), suggesting that the compound interacts weakly with DNA. Derivative **5** resulted to be a strong DNA ligand (Δ*T_m_* = 4.0 °C, 3.0 °C, and 1.5 °C for hp_0_CG, hp_1_CG, and hp_2_CG, respectively). The interaction of **5** with the three DNA duplexes was also monitored at 375 nm, a wavelength at which the ligand absorbs, allowing the monitoring of specific changes related to the ligand (23). Inverted transitions were observed for the three duplexes with the same temperature dependence as that observed at 260 nm. This inverted transition for duplex hp_2 with compound **5** is shown in Fig. 4*D* and is representative of experiments with the other duplexes (data not shown). This transition was not observed with **SGI-1027** and the other compounds (data not shown). This indicates that, upon duplex melting (as observed at 260 nm), the absorbance properties of **5** are concomitantly modified, strongly supporting that **5** interacts with DNA. Hence, the most potent inhibitor of full-length DNMT1 is also the strongest DNA binder (Table 1).

Next, for compounds that interact with DNA, an interesting issue is the selectivity of the compounds toward the DNA sequence. This point could not be addressed upon use of the three hairpin duplexes because they melt at different temperatures. Thus we addressed it by DNase I footprinting analysis.

**FIGURE 5.**
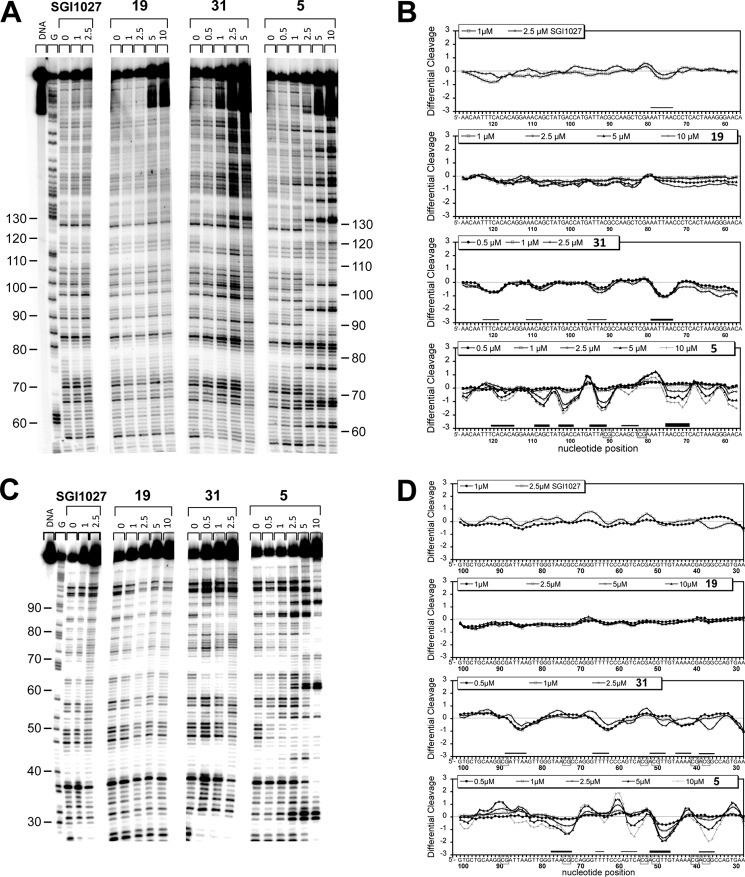
**DNase I footprinting analysis.** 265 bp (*A* and *B*) or 117 bp (*C* and *D*) radiolabeled DNA were incubated with increasing concentration (μm) of the indicated compounds before DNase I digestion and separation of the digested fragments on a denaturing gel (A-C) for further densitometric analysis (*B–D*). *Gray lanes* and *gray boxes* localize footprints and CpG dinucleotides, respectively.

This technique can show where the compound binds on a DNA fragment of a known sequence. Two DNA sequences were analyzed: a 265-bp DNA fragment containing some AT-rich sites and two CpG sites and a 117-bp DNA fragment with several CpG sites in a less AT-rich environment (Fig. 5).

Both DNase I footprinting gels and densitometric analyses confirmed that **SGI-1027** binds weakly to DNA showing some noise (as poor and nonspecific DNA binding) in the differential cleavage pattern, as does compound **19**. Compound **31** partially protected the DNA from DNase I digestion at concentrations up to 2.5 μm, with a preference for A/T stretches on the 265-bp DNA fragment (Fig. 5, *A* and *C*) but shifting to CpG binding (positions 37–38 and 50–51) on the 117-bp DNA fragment. However, the protection was weak. Noteworthy, for both **SGI-1027** and **31**, we could not test higher concentrations than the reported ones as it resulted in cleavage inhibition (smearing), which interfered with sequence selectivity analysis.

Compound **5** clearly showed much stronger footprints surrounded by huge cleavage enhancements as evidenced on the gels by strongly digested bands (Fig. 5, *A* and *C*) and on the densitometric analyses by positive differential cleavage calculated relatively to control *lanes 0* in the absence of the compounds (Fig. 5, *B* and *D*). Clearly, in agreement with the *T_m_* analysis, **5** is the strongest DNA binder of the series showing binding to CpG dinucleotides (positions 37–38 and 50–51, Fig. 5*D*; position 88–89 evidenced at 10 μm, Fig. 5*B*). The strong DNase I cleavage enhancements observed may reflect some distortion of the DNA helix induced by the binding of **5** (24).

Both DNA duplex thermal denaturation and DNase I footprints showed that **19** does not interact with DNA and that **SGI-1027** and **31** display weak interaction. In contrast, **5** is clearly the strongest DNA ligand and the most potent inhibitor of DNMT1. Next, we investigated whether the compounds directly interact with DNMT1 by using thermal shift analysis of the protein unfolding temperature.

##### Compound **5** Interacts with DNMT1 Only When the DNA Is Present

In the fluorescence-based thermal shift assay (differential scanning fluorimetry), the ability of a molecule to stabilize or destabilize the protein during its thermal unfolding is quantified by its thermal shift (Δ*T*_½_): the difference in the protein unfolding temperature in the presence and absence of the ligand.

**FIGURE 6.**
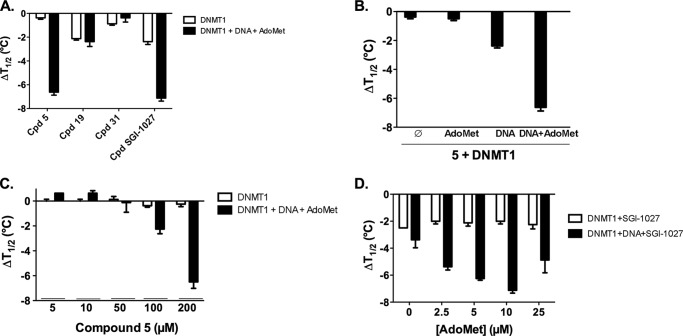
**Thermal shifts analysis of SGI-1027 and its analogs in complex with human DNMT1.** *A*, effect of **5, 19, 31,** and **SGI-1027** incubated at 200 μm with 2.5 μm DNMT1 alone (*white bars*) or in the presence of 5 μm AdoMet and of DNA (*black bars*). *B*, effect of the AdoMet and DNA substrates. For each condition, DNMT1 was incubated at 2.5 μm with 200 μm compound **5**, and 5 μm AdoMet and/or 5 μm DNA duplex. *C*, concentration effect of **5** (5–200 μm). DNMT1 alone at 2.5 μm (*white bars*) or in the presence of 5 μm AdoMet and DNA (*black bars*) was incubated with increasing concentrations of compound **5**. *D*, effect of increasing concentrations of AdoMet (from 2.5 to 25 μm) on 2.5 μm DNMT1 alone (*white bars*) and in the presence of 5 μm DNA (*black bars*) incubated with 200 μm **SGI-1027**. The means of at least two experiments are displayed with the corresponding S.E.

The DNMT1 unfolding temperature was determined to be 46.8 ± 0.1 °C in absence of any ligand. No variation or Δ*T*_½_ of 3.4 ± 0.2 °C was observed when DNMT1 was incubated with 5 μm AdoMet or 5 μm DNA, respectively (data not shown). Finally, in the presence of both DNA and AdoMet, the Δ*T*_½_ was 6.1 ± 0.4 °C suggesting a cooperation between the two ligands (data not shown). In the presence of 200 μm inhibitor **5** (Fig. 6*A*) no variation was measured in the absence of DNA and AdoMet; in contrast a strong destabilization was observed when the DNA and AdoMet were present in the solution (Δ*T*_½_ = −6.6 ± 0.2 °C). Thus compound **5** did not directly interact with DNMT1 but needed the formation of the DNMT1-AdoMet-DNA enzymatic complex. This observation was confirmed when each substrate was added separately (Fig. 6*B*). In fact, a variation in the *T*_½_ of DNMT1 was only observed when the DNA duplex was present in addition to compound **5**. Noteworthy, the destabilization was strongest when AdoMet was also present. In addition, a dose-response curve was obtained for the Δ*T*_½_ of the enzymatic complex with the increase in compound **5** concentration (Fig. 6*C*). Similar results were obtained on DNMT3Acat (data not shown).

The other compounds interacted weakly with DNMT1 alone or in the presence of its two substrates (DNA and AdoMet), with the exception of **SGI-1027**, which showed a strong destabilization in the presence of the ternary complex (Δ*T*_½_ = −7.1 ± 0.2 °C, Fig. 6*A*). Compounds **19** and **SGI-1027** presented a weak destabilizing effect on DNMT1 alone (Fig. 6*A*) that could be observed also on the ternary complex.

Noteworthy, in agreement with the AdoMet non-competitive behavior observed in the enzymatic studies for **SGI-1027**, the destabilization of the DNMT1 and of the DNMT1-AdoMet-DNA complexes was little dependent on the AdoMet concentration (Fig. 6*D*). The slight effect observed mainly arises from the stabilizing effect of increasing concentration of AdoMet on the DNMT1-DNA complex (data not shown).

##### Compound **5** Destabilizes the DNMT1-DNA Complex

**FIGURE 7.**
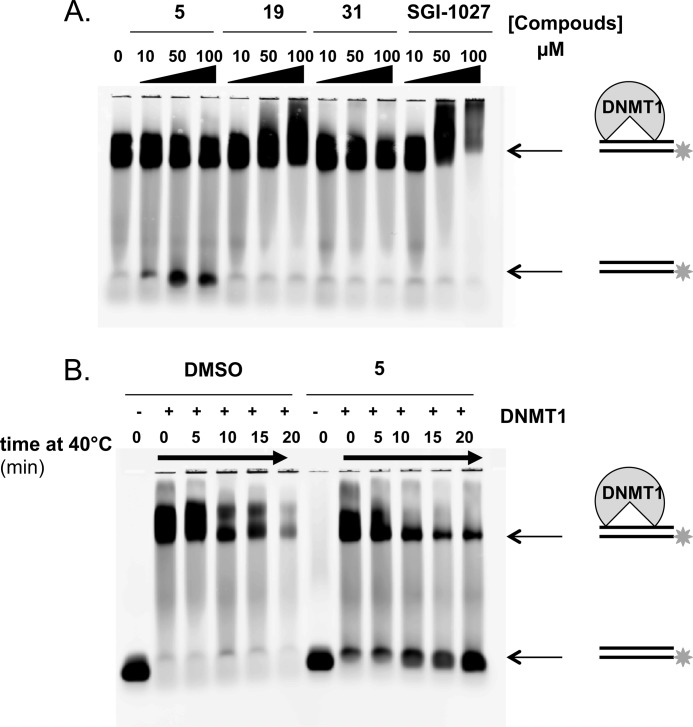
**Electrophoresis gel shift assay of DNMT1 complexed to DNA.** *A*, effect of the addition of increasing concentrations of **5**, **19**, **31**, and **SGI-1027** (from 10 to 100 μm) to 2.5 μm DNMT1 in the presence of 5 μm AdoMet and 0.5 μm fluorescein-labeled DNA duplex. *B*, time course experiment upon heat denaturing at 40 °C of the complex formed between 2.5 μm DNMT1 and 0.5 μm fluorescent DNA duplex with 5 μm AdoMet in the absence and in the presence of 100 μm compound **5**.

To further explore the destabilization of the enzymatic complex by the compounds, DNMT1 was complexed on a fluorescein-labeled DNA duplex in the absence or in the presence of the different compounds and migrated on an agarose gel (Fig. 7*A*). Only compound **5** destabilized the DNMT1-DNA complex with the appearance of the free duplex as the concentration of the compound increased. In addition, heat denaturing experiments at 40 °C showed that the DNMT1-DNA complex is dissociated faster in the presence of compound **5** (Fig. 7*B*).

## DISCUSSION

Our data indicate that **SGI-1027** and its two analogs, **5** and **31**, are inhibitors of DNMT3Acat and DNMT1. Compound **5** is the most potent inhibitor of DNMT1 among the three. These compounds behaved as AdoMet non-competitive inhibitors of DNMT1 and, rather, displayed non-Michaelian behaviors in DNA competition studies on DNMT1, which is characteristic of DNA competitive inhibition with DNA-inhibitor interactions (17). These results are in disagreement with previous reports describing a AdoMet competitor behavior for **SGI-1027** (9, 10). Datta *et al.* (9) analyzed only parts of the velocity plots against AdoMet concentration; a plateau was not reached in their experimental conditions rendering the interpretation more difficult. Fagan *et al.* (10) conducted competition experiments on a truncated form of DNMT1 (residues: 621–1600 amino acids) missing the replication focus targeting sequence (RFTS) domain. Importantly, this domain was previously described to be an endogenous DNA competitive inhibitor of DNMT1 (25) and to bind to the DNA binding pocket of DNMT1 (26). Thus, it is not surprising that DNA competition studies on truncated DNMT1 are different from ours carried out on the full-length enzyme. Regarding the deviations from the Michaelis-Menten model observed in our DNA competition experiments, they can also result from other causes than substrate-inhibitor interactions. For example, they can be the consequences of tight binding or time-dependent inhibition. We cannot exclude these other hypotheses. However, we experimentally observed that the compounds interact with DNA and in particular compound **5**, supporting this inhibition mechanism. Indeed compound **5**, the most potent inhibitor (Table 1) on DNMT1, is a strong DNA binder (Table 1 and Fig. 4), whereas **SGI-1027** and **31** interact only weakly. These derivatives present a preference for A/T stretches, G/C-rich stretches, and certain CpG sites in the 117-bp DNA fragment studied here (Fig. 5). Interestingly, the change from the *para* to the *meta* bonds from **SGI-1027** to **5** increased the DNA binding properties, suggesting a better fitting in the DNA groove (27). Finally, for compound **5**, we were able to establish that this molecule only interacts with DNMT when the DNA is present (Fig. 6) and induces a destabilization of the DNMT1-AdoMet-DNA complex (Fig. 7). **SGI-1027** was also shown to be able to destabilized the DNMT1 in the presence of DNA and AdoMet by differential scanning fluorimetry (Fig. 6).

In conclusion, both enzymatic assays and biophysical studies indicate that the most potent inhibitor of DNMT1, compound **5,** inhibits DNMT by interacting with DNA and destabilizing the enzymatic complex.

## Footnotes

3 The abbreviations used are:

DNMT: DNA methyltransferase
AdoMet: *S*-adenosyl-l-methionine
AdoHcy: *S*-adenosyl-homocysteine.
